# Empowering patients in primary care: a qualitative exploration of the usability and utility of an online diabetes self-management tool

**DOI:** 10.1186/s12875-024-02358-9

**Published:** 2024-04-11

**Authors:** Jeremy Wei Song Choo, Aminath Shiwaza Moosa, Jeremy Wei Mei Koh, Chirk Jenn Ng, Ngiap Chuan Tan

**Affiliations:** 1 SingHealth Polyclinics, Singapore, Singapore; 2 SingHealth-Duke NUS Family Medicine Academic Clinical Programme, Singapore, Singapore

**Keywords:** Diabetes mellitus, type 2, Patient education, Primary health care

## Abstract

**Background:**

Despite the potential advantages of Internet-based diabetes self-management education, its adoption was not widespread among Singapore’s public primary care clinics (polyclinics). An interactive online tool was thus developed to help educate patients with Type 2 diabetes mellitus (T2DM), and was now ready for user testing before implementation.

**Aim:**

To explore the perceived utility and usability of the educational tool in patients with suboptimally-controlled T2DM in a Singapore primary care setting.

**Methods:**

In-depth interviews were used to gather qualitative data from multi-ethnic Asian adults who had suboptimally-controlled T2DM. A total of 17 IDIs were conducted between April 2022 to March 2023, audio-recorded, transcribed, and analyzed to identify emergent themes via thematic analysis.

**Results:**

Regarding utility, users found the educational tool useful because it provided them with information that was comprehensive, accessible, reliable, and manageable. Regarding usability, the majority of users reported that the educational tool was easy to use, and suggested ways to improve navigational cues, visual clarity, readability and user engagement.

**Conclusion:**

Participants generally found the educational tool useful and easy to use. A revised educational tool will be developed based on their feedback and implemented in clinical practice.

## Introduction

Type 2 diabetes mellitus (T2DM) is widely recognized as a major contributor of premature deaths from non-communicable diseases globally. Its associated social and financial implications are of growing concern to the international community [1]. In response, the World Health Organization has prioritized controlling T2DM [2]. Similarly, Singapore has declared “War on Diabetes” in 2016, implementing national policies to prevent, detect, and manage this condition [3].

At an individual level, effective management of T2DM requires patients to make informed decisions regarding their diet, physical activity, and adherence to medication regimens. Diabetes self-management education (DSME) is a crucial component in facilitating this process. Multiple studies have shown that DSME leads to improved patient knowledge, self-care practices, glycaemic markers, and lower complication rates [4–5].

The delivery of DSME through the Internet has shown comparable outcomes [6–7], with added benefits of easier access, greater reach, and better cost-effectiveness [8–9]. Patients are able to learn at their own pace without time constraints, and can revisit educational materials as needed [10]. With the growing use of telemedicine amidst the COVID-19 pandemic [11–12], online educational tools can also be incorporated into remote consultations to promote shared decision-making and reinforce learning [13].

Despite the potential advantages of Internet-based DSME, its adoption was not widespread among Singapore’s public primary care clinics (polyclinics), which have the responsibility of managing a significant portion of the diabetic population in the country [14]. This represents a missed opportunity given that Singapore has one of the highest penetrance of info-communication technology in the world [15]. Moreover, a study by Asharani et al. showed that the local population had high functional health literacy, indicating an ability to understand and respond adequately to healthcare communications [16].

Although polyclinics do not actively deliver DSME through the Internet, a considerable number of Internet users, especially those with chronic illnesses, utilize it as a source of health information [17–18]. However, many of these individuals face difficulties in finding needed information and are uncertain about its reliability [19]. Furthermore, even when accessing dependable sites such as WebMD or the American Diabetes Association website, patients frequently encounter information overload, resulting in confusion [20].

Besides informational hurdles, local patients have pinpointed poor IT literacy as the principal barrier hindering their participation in IT-based health education [21]. In response, SingHealth Polyclinics, a cluster of public primary care clinics in Singapore, initiated the development of a learning tool catered to the specific needs of the local context. In collaboration with Universiti Malaya and Universiti Putra Malaysia from Malaysia, an interactive online educational tool, known as a Reusable Learning Object (RLO), was developed to help patients with suboptimally-controlled T2DM “understand the options that can be taken to better manage their diabetes” [22]. The RLO presents practical and concise information that is tailored to the local practice setting, and provides guidance on both non-pharmacological and pharmacological management options. Additionally, it incorporates a range of media such as illustrations, diagrams, narrations, and videos to improve the user experience.

There have been numerous studies on technology-assisted DSME, providing insights into the information patients require and the most effective means of conveying it. The developers were mindful of these considerations during development. The next step would be to engage patients with T2DM to actively test the tool and provide feedback on its utility and usability [23]. Utility measures the RLO’s usefulness in helping users achieve the learning objective [24], whereas usability assess its ease of use [25]. The aim of this study is to explore the perceived utility and usability of the RLO in patients with suboptimally-controlled T2DM in a Singapore primary care setting. The findings from this study will help improve the RLO before its implementation in clinical practice.

## Methods

### Study design

A qualitative research methodology was chosen to explore participants’ perceptions of the utility and usability of the RLO. This approach was favoured for its ability to provide rich insights into the “how” and “why” aspects of user experience, rather than solely focusing on the “what” [26]. In-depth interviews (IDIs) were conducted to gather participants’ views, clarify their perspectives, and solicit suggestions for improvement. Grounded in a descriptive-interpretative framework [27], this approach was well-suited for examining subjective phenomena. Researchers sought to capture participants’ perceptions, shaped by their individual experiences and social contexts, and subsequently engaged in interpretation using an inductive and iterative approach to reveal valuable insights.

### Study site

The study site was Sengkang Polyclinic, a public primary care clinic located in north-eastern Singapore. The polyclinic provides comprehensive primary care services to about 300,000 multi-ethnic Asian residents, and serves an average of 900 to 1000 patients on a typical workday. Notably, the clinic attends to around 13,000 patients with T2DM, which results in approximately 43,000 attendances per year.

### Study population

The target participants were multi-ethnic Asians, aged 21 years and above, who had suboptimally-controlled T2DM (latest HbA1c reading above 8.0%, as reflected in the polyclinic electronic medical records). As the RLO was only available in English, participants should be able to read and speak English, and must have a self-reported ability to access and navigate websites. Patients with any disability or impairment which rendered them incapable of providing informed consent were excluded.

### Recruitment

The investigators (CWSJ and KWMJ) identified eligible patients during clinical consultations and invited them to participate in the study. They were reassured that there would be no impact on their care should they refuse. Those who were interested were directed to the study team’s Clinical Research Coordinator (CRC) who gave them a participant information sheet to read, explained the study in detail, and answered any queries. Patients who decided to join the study were recruited after they provided written informed consent.

Recruited participants then completed a questionnaire to provide demographic data and details on their T2DM condition. They were also given the website linked to the RLO as well as the topic guide, with the instruction to use the RLO and formulate their opinions about it. The interview was scheduled at least one week later so as to allow adequate time for the participant to review the materials thoroughly.

The recruitment and IDIs took place from April 2022 to March 2023. Purposive maximum variation sampling was carried out to include participants of diverse age groups, education levels, and durations of T2DM to gather a wide range of perspectives. Data collection continued until data saturation was reached.

### RLO

Clinicians from Singapore and e-learning experts from Malaysia jointly developed the RLO, with the former contributing clinical content and the latter providing pedagogical input and technical support. The development process was guided by the ASPIRE framework [28] from the University of Nottingham, UK, and Table 1 outlines the content and design features of the RLO.

**Table 1:** RLO content and design features

Section	Content	Design features
Cover page	Learning objective of the educational tool	- Illustration showing 4 options to improve diabetic control
Introduction	User indicates if their diabetic control is good or bad	- Flipcharts
What are your options	Brief explanation of the options to improve diabetic control	- Flipcharts
Option 1: Exercise	Exercise recommendations, as well as the pros and cons of exercise	- Colour-coded table - Audio narration
Option 1: Exercise (continued)	FAQs on exercise	- Flipcharts
Design your own exercise programme!	A sample exercise program, which incorporates a variety of exercises over the course of a week	- Flipcharts
Option 2: Diet (A sample view is shown in Fig. 1.)	Dietary recommendation, as well as the pros and cons of diet control	- Information arranged in a colour-coded table - “My Healthy Plate” – user can hover the mouse over numbered buttons to see how to fill each section of the plate.
Option 3: Insulin (A sample view is shown in Fig. 2.)	Information on insulin, including types, usage, as well as pros and cons (such as side effects)	- Colour-coded table - Video with narration
Option 4: Oral Medications	Pros and cons of different classes of oral medications for T2DM	- Colour-coded table - Video with narration
What is your decision?	User indicates if they are ready to make a decision to control their diabetes, and if so, which option(s) they prefer	- Video with narration
Summary	Restating the learning objective, as well as links to related educational tools	- Hyperlinks

**Fig. 1:**
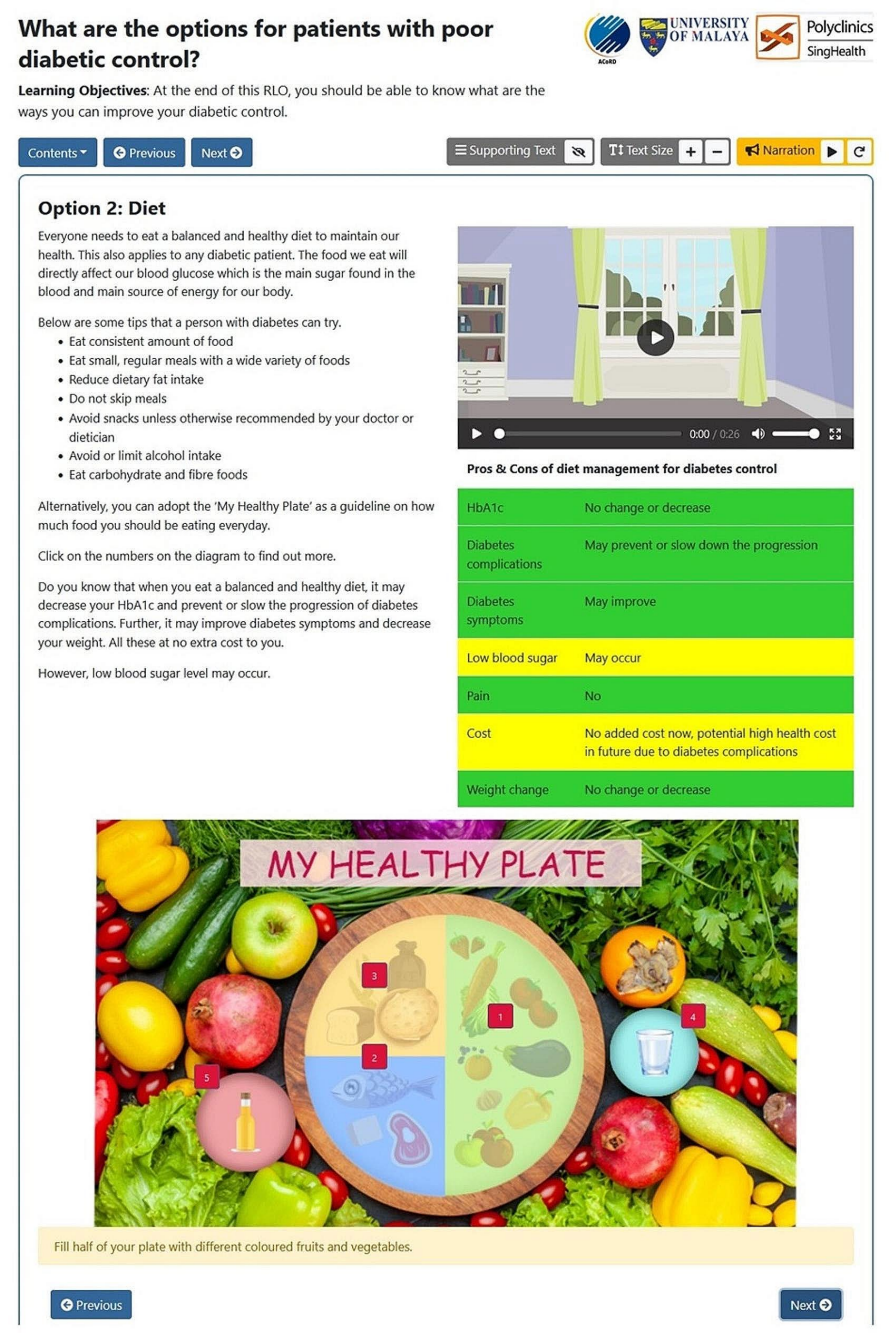
Sample view of the RLO (Option 2: Diet)

**Fig. 2:**
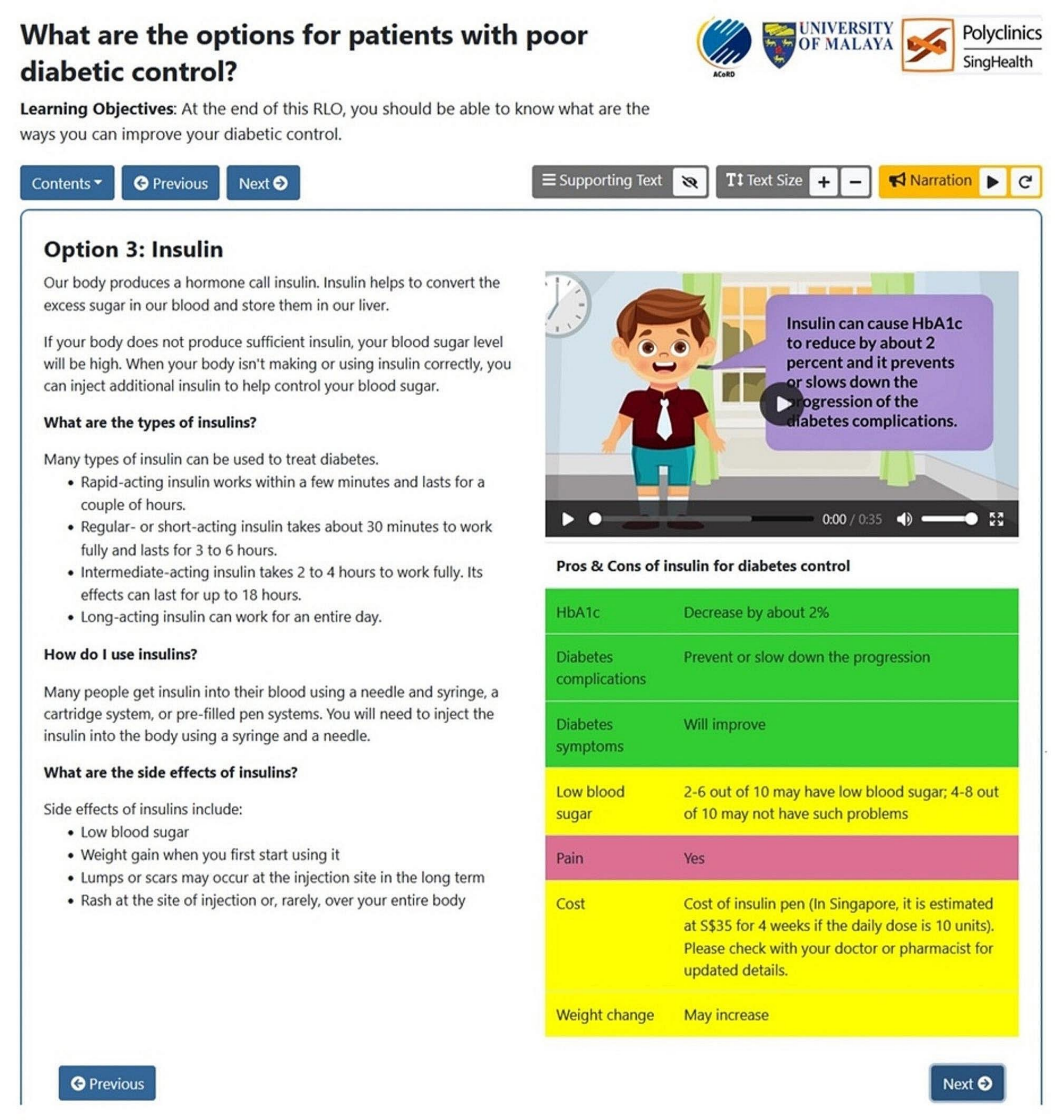
Sample view of the RLO (Option 3: Insulin)

### Topic guide

A semi-structured topic guide was developed based on the Technology Acceptance Model [29], literature review, and discussions between study investigators. The key questions asked during the interviews are listed in Table 2. Flexibility was embraced in the interview process, allowing the interviewer to adapt to the flow of the conversation. This involved posing follow-up questions to explore issues, probe for details, and seek clarification. The questions were refined as more interviews were conducted, and sub-questions were added to explore emerging themes.

**Table 2:** Topic guide with the key questions

1	How was your experience using the educational tool to learn about diabetes management?
2	Was it useful? Please explain. (Perceived Usefulness)
3	Was it easy to use? Please explain. (Perceived Ease of Use)
4	We may want to make this educational tool widely available to our patients with diabetes. What are your views on that? (Attitude Toward Using, Behavioural Intention to Use)
5	We may want to develop other web-based educational tools, to help educate our patients about their illnesses. What are your views on that?
6	Do you think it is better to use these tools before or after seeing the doctor? (Implementation)
7	Is there anything else you would like to share?

### Interviews

The principal investigator, CWSJ, conducted all the interviews and took field notes. He is a family physician with a Masters in Family Medicine and works in Sengkang Polyclinic where the study was conducted. He has attended qualitative research training and workshops, and was closely supervised by two experienced researchers (NCT, CJN) who provided feedback through direct observation of the interviews and by listening to the audio recordings.

CWSJ was not involved in the development of the RLO and had thoroughly familiarized himself with the research topic and relevant literature. During the pre-interview briefing, voluntary participation, confidentiality, and frank opinions were emphasized. Participants were anonymized and addressed by their study identification. The interviews were conducted individually, primarily face-to-face, in a quiet room in the polyclinic to ensure privacy. However, participants who preferred an online interview were offered the option of using Zoom, an online teleconferencing platform. CWSJ strived to adopt a neutral stance throughout the IDIs. He practised active, empathetic active listening, and paid close attention to the participants’ responses and non-verbal cues. Ample time was provided for participants to freely share their personal views, whether positive or negative, without interference or coercion.

Participants were reimbursed with grocery store vouchers of SGD20 (approximately USD15) for their time and travel expenses. The interviews were audio-recorded and transcribed verbatim by a professional transcriber. CWSJ then audited the transcribed texts with reference to audio recordings, corrected errors, and used the edited transcripts as data for analysis.

### Data coding and analysis

Data collection and analysis occurred concurrently. CWSJ and ASM read and re-read the first two audited transcripts for familiarization, then coded them independently. They did this via an inductive approach, labelling units of data, by phrases or lines, with codes. Inter-rater reliability was not formally assessed, but there was a high level of agreement between the coders when they convened to discuss the accuracy of their codes. The few differences were deliberated upon, and consensus was reached to form an initial coding frame.

This coding frame was then applied to the subsequent transcripts, which were coded by CWSJ using NVivo software. As more interviews were completed, new codes surfaced, and the coded data were inductively grouped into emergent themes [30]. Having a single coder ensured consistency in coding decisions across transcripts, yet it also introduced the risk of bias. To address this potential issue, CWSJ applied the principle of reflexivity, conducting coding and analysis in a reflective and iterative manner. Researcher triangulation was additionally employed through regular discussions among the research team, during which they delved into the codes and themes to improve relevance, clarity, and depth. Refinements were then made after each deliberation.

The interviews were terminated after 17 IDIs as data saturation was deemed to have been reached, despite repeated reviews. Data saturation is defined as the point when no new code emerged. The consent forms, participant details, questionnaires, audio recordings, field notes, transcripts and coding were all organized in secure archives to ensure a clear audit trail.

## Results

Seventeen participants were interviewed, consisting of eight men and nine women from different ages (ranging from 36 to 65), ethnicities, education levels, and durations of T2DM. The details are shown in Table 3. Of the 17 IDIs conducted, 16 were held in-person, and one was conducted over Zoom, each lasting between 24 and 60 min. 3 participants who initially consented to the study dropped out due to scheduling difficulties.

**Table 3:** Demographic characteristics of the study population

Demographic characteristics	Participants (*n* = 17)	Mean (where relevant)
Gender		NA
Male	8	
Female	9	
Age (years)		52
30–39	1	
40–49	6	
50–59	6	
60–69	4	
Ethnicity		NA
Chinese	7	
Malay	6	
Indian	3	
Others: Filipino	1	
Education		NA
Primary	2	
Secondary	4	
A-level / Diploma	7	
University / Post-tertiary	4	
Employment status		NA
Homemaker	1	
Employed	14	
Unemployed	1	
Retired	1	
Duration of T2DM (years)		9
1–5	4	
6–10	8	
11–15	3	
More than 15	2	

As per the study aim, the results were categorized under Utility and Usability, with the former assessing the information’s usefulness, and the latter examining its delivery. Under each of this broad category, themes and sub-themes were reported and supported by quotations from the interviews.

### Utility

#### Comprehensiveness of information

Participants, even those with more experience and prior knowledge of T2DM, were able to acquire new information about their condition.

*“As a diabetes patient, many times I am not sure what to ask. But this gives a lot of new information, on weight management, different types of medicine.” (Participant 1, 41 years old (y.o.))*.

They wanted in-depth knowledge about the management options for T2DM, which some felt was not adequately explained during clinical consultations.

*“I want to know what I take. Don’t just blindly, people ask you to eat, you just pop, pop, pop, pop. Medications are good to help improve your health, but at the same time, they can have side effects.” (Participant 6, 44 y.o.)*

*“This is more in-depth because we know what are the medications that they’re giving and what kind of exercise we are supposed to do, how many minutes or hours. I find this more informative [than a doctor’s consult]. If we ask them, they will tell. Otherwise, they will just as per normal say, ‘Oh, you have this reading, so I prescribe you this medication.’” (Participant 12, 52 y.o.)*

Users appreciated it when the RLO provided adequate and granular details as they could apply the information to self-manage their diabetes.

*“This is good because you specify which kind of exercise is good, how many minutes, so people will know ‘Oh this will impact my diabetes. I can do this exercise as well.’” (Participant 12, 52 y.o.)*

*“Better to give more examples. The information is too little. Maybe you can do it like the exercise part, day by day, more guidance.” (Participant 1, 41 y.o.)*

*“I think it will be better to list the foods that diabetic patients should avoid. I always go to the supermarket for grocery shopping, so I have to know which ones I can buy, which ones I cannot buy.” (Participant 5, 47 y.o.)*

Specifically, they asked for examples that were tailored to the local context.

*“A long time ago, Health Promotion Board (HPB) produced this booklet that had all the calorie counts. I suggest you link into that one because that is local food. Otherwise, if you go into ‘Ang Moh’ (Caucasian) websites, they always eat spaghetti, so everyone is just going to roll eyes.” (Participant 14, 50 y.o.)*

#### Accessibility of information

Participants found it more convenient to access the RLO online as compared to printed materials.

*“I think the web is better. Because hardcopy, when people take, after that they put the paper one side, don’t bother to read. But everybody will be using their phone, 24 hours.” (Participant 3, 36 y.o.)*

They shared that it was useful to be able to revisit and review the material whenever necessary.

*“Over the years, you forget what was advised to you. With this, you can always go back and check.” (Participant 2, 43 y.o.)*

*“Actually, see doctor 2–3 months once, right? So, if I got problem, I don’t know where to find, how to do… So, when I refer this one, they tell me, ‘If you are like this, you can do like this, like that’. So, at least I can get more knowledge through this.” (Participant 16, 58 y.o.)*

#### Reliability of information

Users trusted the RLO’s information more than that from family, friends, or other online sources.

*“Family and friends – you must know from which sector they are in. Not everybody will give you the right information.” (Participant 12, 52 y.o.)*

*“Google is Google okay. Anybody can say anything.” (Participant 15, 57 y.o.)*

They deemed it trustworthy because it was developed and endorsed by the Polyclinic.

*“I feel on the internet there are so many things. Different viewpoints, so which is the correct one – you don’t have anything authenticated… This one at least, polyclinic knows me, I know the polyclinic, and we are working it out together.” (Participant 10, 59 y.o.)*

*“Because I see even the logo is there, the polyclinic logo. If it has an official logo and things like that, it gives you some real good feeling. You are not going into the wrong information.” (Participant 15, 57 y.o.)*

However, some users highlighted instances of information inconsistency.

*“On the item that says cost “No added cost”, it should be highlighted in green because there’s no added cost. It shouldn’t be highlighted yellow… Under “Sodium-glucose transporter”, the table says, ‘45 dollars per week’, but the video says, ‘50–130’, which is quite a big difference.” (Participant 14, 50 y.o.)*

#### Manageability of information

Participants considered the information relevant to the learning objective and appropriate in volume.

*“When I want to google certain things, different websites link here and there, a lot of information. You may not want that information, but it is there.” (Participant 12, 52 y.o.)*

*“Googling this kind of things sometimes may give you too much information you know. Everybody will become your doctor… This one is just nice, and you know what you need to have.” (Participant 15, 57 y.o.)*

#### RLO as a decision tool

There were mixed reactions to the final section of the RLO, where users were asked to make a decision regarding their preferred management option(s). Some viewed commitment as helpful, but others felt pressured.

*“This page is good. At last, they ask you ‘Are you in it? Do you want to make a change for yourself?’ It is your final statement to make a change and improve.” (Participant 6, 44 y.o.)*

*“Nobody wants to tell you what they decide. When you ask me a decision, I may not even want to reply. I feel like I am being pressured.” (Participant 8, 61 y.o.)*

### Usability

#### IT-savviness needed to navigate RLO

Overall, most participants found the RLO to be user-friendly.

*“Easy to use, not only for youngsters, but even for the elderly, if they know how to use the handphone” (Participant 3, 36 y.o.)*

Some users with lower IT literacy faced challenges, but they expressed a willingness to learn.

*“I am not very comfortable with online, but it is better to learn… Sometimes when I am at home and I don’t know, I will ask my daughter, I ask my grandchildren. They teach me. If not, I also don’t know how to use this handphone Apple one. Last time only Nokia. For me, it is okay. Like I said, must learn. Because the government now says, all must learn digital, better.” (Participant 7, 63 y.o.)*

Clearer labels were requested by a few users as it was not apparent to them that they needed to click on some of the RLO features, such as the flip-charts and audio icon.

*“For people who play with a lot of tabs and they know about this, no problem they just tap and they find out, especially kids today. But for my age, I look at it, I may stop there, I may not even tap.” (Participant 8, 61 y.o.)*

*“I didn’t know there was something there. When I click, there was a voice coming out… Got to put some signage on this. Otherwise, people won’t know. I thought it was just reading material.” (Participant 12, 52 y.o.)*

#### Visual clarity

The majority of users perceived the visuals of the RLO to be clear, although some areas for improvement were identified. One user felt that the default font size was too small, but appreciated the feature to increase it. Other users suggested enlarging the click-buttons and enhancing the contrast of the pictures.

*“If you have so much information for me, please create a contrast so that I can still read what you need me to do.” (Participant 8, 61 y.o.)*

#### Simplicity of language

The language used in the RLO was deemed appropriate and easy to understand by most of the participants.

*“It is short, simple, and easy to understand, that’s why I like it. So far, nothing is challenging in this because it is down to layman’s terms.” (Participant 12, 52 y.o.)*

However, those with a lower level of English proficiency struggled with more advanced vocabulary, leading to recommendations to translate the RLO into other commonly-used languages in Singapore.

*“Okay this one: ‘moderate’. This is the meaning that I don’t understand. Because for me, I studied only until Primary 6, that’s why some of the meaning I not sure.” (Participant 4, 43 y.o.)*

*“Because Malay version for my age, better… Actually, it’s simple English. Not really hard. But sometimes 1 or 2 words make me like ‘What is this? What is this?’” (Participant 16, 58 y.o.)*

Furthermore, some users pointed out the use of medical jargon that was hard to comprehend.

*“HbA1c? You don’t describe it here. So, if I’m not a medical person, I may not understand. At least put it in the footnotes.” (Participant 11, 65 y.o.)*

#### Positive language

Besides using uncomplicated language to facilitate conveyance of information, the importance of employing positive language was highlighted. One user considered the phrase “poor diabetic control” to be judgmental.

*“At the top, ‘What are the options for patients with poor diabetic control’. Maybe you should make it more positive, just say, “Options for better diabetic control”. So the person who is reading it don’t feel like, ‘Wah, judging me’… Make it less judgmental. Don’t use ‘Bad’, instead ‘Can be better’, or ‘Can be improved’. (Participant 14, 50 y.o.)*

Although this opinion was not widely shared, others also brought up the impact of positive language on emotions and motivation.

*“This question, ‘How is your diabetic control?’. I just click ‘Bad’ because I know myself, so the tool will say, ‘Don’t be disheartened.’ It gives me comfort, gives me encouragement to take care of myself, unlike my husband – he always scolds me for not listening to him. (Participant 13, 49 y.o.)*

#### Preference for multimedia over text

The participants indicated that the incorporation of multimedia features increased engagement and interactivity.

*“It is not boring because they use a lot of animation, and also when you press, there are a lot of notes at the back. It is kind of interactive.” (Participant 5, 47 y.o.)*

However, a few of them felt the RLO was too wordy, and proposed the inclusion of more visuals, such as pictures and videos.

*“I think you all should have more pictures, not just words to describe. When you read, especially my age, if it’s too much, your eyes get tired very easily.” (Participant 11, 65 y.o.)*

*“Animate it, make it a bit more enjoyable. Because the whole thing is like read, read, read.” (Participant 12, 52 y.o.)*

Users who found reading demanding welcomed the audio feature, although a few commented on its slow pace.

*“I’m the type of person that seldom read all the page until finish. That’s why it’s good to have audio.” (Participant 3, 36 y.o.)*

*“If I don’t understand, I can repeat and repeat [the audio], listen to all the meaning… Words, I need to wear specs, then I need to remember again.” (Participant 4, 43 y.o.)*

*“I played the narration because I was just relaxing and I just want to hear. But it was a bit too slow, so I just paused and read.” (Participant 2, 43 y.o.)*

#### Repetitiveness in the RLO

Finally, it was noted by users that some parts of the RLO was unnecessarily repetitive, causing them to lose interest.

*“Okay so page 10 right, under the ‘Yes, I’m ready’, every option that I click gives the same message. So I got a bit like roll eyes after the second click. So, unless you like change the words, one says ‘Good’, one says ‘Fantastic’, one says ‘Good job’ you know.” (Participant 14, 50 y.o.)*

## Discussion

This study contributed to the existing knowledge on Internet-based DSME by reinforcing the importance of established best practices, as well as demonstrating the benefits of user testing in identifying gaps in the development process. Regarding utility, users found the educational tool useful because it provided them with information that was comprehensive, accessible, reliable, and manageable. Regarding usability, the majority of users reported that the educational tool was easy to use, but they also gave feedback to improve the delivery of information by improving navigational cues with clearer labels, enhancing visual clarity with larger click-buttons and stronger contrast, using simple and positive language, employing more visual content to reduce verbosity, and avoiding repetition to sustain user engagement.

In their qualitative systematic review on technology-assisted DSME, Jain et al. found that patients with diabetes wanted health information in order to gain a sense of control over their disease [31]. Locally, patients preferred to obtain this information through face-to-face sessions rather than IT-based methods [32]. However, effective face-to-face education often necessitates prolonged contact time which may not be feasible in a polyclinic setting with high patient loads, long waiting times, and consultation durations averaging 10 min per patient [32]. Consequently, most patients receive only one health counselling session at the onset of their T2DM diagnosis, with no further formal counselling thereafter. The results of this study showed that the RLO added value by being an adjunct to the clinical consultation, allowing in-depth patient education to take place beyond the consultation room, whenever and wherever there is Internet access.

However, the lack of examples on local food choices in the Diet section was identified as an information gap by users. This aligns with the Sohal et al’s research on the barriers of T2DM management in South Asians, which emphasized the need for culturally-tailored diabetic education, proposing that dietary advice should provide concrete examples of Asian foods and ingredients, along with details on portion size and cooking methods [33]. Utilizing such an approach could improve the relevance of the education, resulting in greater effectiveness and improved health outcomes [34–35]. Therefore, the authors intend to work with the development team to integrate more detailed and contextualized lifestyle advice into the RLO.

One of the main benefits of the RLO, according to participants, was its perceived trustworthiness. Trust is a crucial aspect of health education, and research has demonstrated that higher levels of trust are associated with better treatment adherence, glycaemic control, and health-related quality of life [36–37]. In his systematic review on the antecedents of trust in health information websites, Kim et al. highlighted several criteria, of which two are particularly relevant to this study [38]. The first is perceived reputation, where patients tend to trust online information sources operated by reputable organizations. The current study affirms this finding, with users reporting confidence in the RLO due to its affiliation with SingHealth Polyclinics, an advantage that can be leveraged to create more “in-house” educational tools. The second criterion is information quality, which the authors will work to improve by addressing the inconsistencies identified, and ensuring that the information is accurate and up-to-date.

Even though the information presented in the RLO was well-received and deemed reliable by users, some expressed dissatisfaction with the decision-making feature, as it appeared to diverge from the stated objective of the RLO, which was to educate users on ways to improve diabetic control. Despite providing information on management options, the RLO lacked features to help clarify values or guide deliberation, which are recommended by the International Patient Decision Aids Standards Collaboration [39]. To prevent distraction and maintain the RLO’s educational focus, the decision-making component may need to be removed in the next version.

In terms of usability, users provided numerous suggestions to improve navigational cues, visual clarity, readability and user engagement. Many of these ideas are well-established and reiterated in the literature. Jain et al., for instance, has highlighted that patients prefer easy navigations, and appreciate concise information free from medical jargon [31]. Additionally, multiple professional organizations recommend using universal health literacy precautions, such as writing in plain English and utilizing visual aids, to ensure that all patients can understand the information [40]. Despite the developers’ efforts to adhere to best practices, users still identified areas for improvement. What may appear simple to developers may not be simple enough for users. Hence, involving patients in usability testing becomes imperative for a comprehensive evaluation. The use of in-depth interviews, rather than questionnaires, was helpful to pinpoint important informational and navigational issues that were then explored more deeply [41]. The insights garnered will be applied to enhance the subsequent iteration of the RLO.

In line with AshaRani’s study [21], lower IT proficiency correlated with increased difficulties in using the RLO. Nevertheless, most of them expressed a willingness to learn, indicating that this obstacle can be surmounted. Efforts must be made to ensure equitable access to the benefits of digital healthcare services. This can be achieved by developing user-friendly educational tools, refining them based on patient feedback, increasing access to digital devices, and offering technical support and training as required [42–43].

One salient finding that surfaced from the analysis was the importance of presenting information in a positive manner. According to Guo et al., one in six local patients with suboptimal glycaemic control experienced diabetic-related distress, a negative emotional state arising from the burden of living with the disease [44]. The use of judgmental language by healthcare professionals can aggravate this problem. Opting for terms like “suboptimal” instead of “poor control” is recommended as it avoids moral connotations [45]. The next iteration of the RLO will incorporate empathetic and encouraging language to promote empowerment and improve the user experience.

## Conclusion

Through this study, the authors have gained a deeper understanding of the RLO’s utility and usability. Users found the RLO useful because it provided them with information that was comprehensive, accessible, reliable, and manageable. They also found the RLO to be generally easy to use, but gave feedback to improve its navigation, visual clarity, language use, and user engagement. The insights gleaned from the research will be utilized to fine-tune the RLO’s content and design before its implementation in clinical practice.

### Limitations

- Patient selection was limited to English-speaking individuals because the tool was only available in English, though the plan is to translate it to other languages in future so as to benefit more patients.
- The study did not include patients from private clinics, who may have distinct demographics and healthcare experiences that could have influenced their views on the RLO’s utility and usability.
- Member checking would have helped ensure appropriate interpretation of the qualitative data, but was not carried out due to the constraints of the study timeline.

## Data Availability

The data and other related material are available upon request to the corresponding author.

## Declarations

### Patient and public involvement

Prior to commencing interviews, the research question and protocol were presented to a group of volunteer patients. Their perspectives on the project’s value, safety, and ethics were meticulously assessed and duly taken into consideration.
